# Comparison of clinical characteristics, microvascular complications and inflammatory markers in type 2 diabetic patients under insulin versus metformin treatment: A cross-sectional study at Karbala Diabetic Center, Iraq

**DOI:** 10.1097/MD.0000000000040330

**Published:** 2024-11-01

**Authors:** Haithem Rauf Mohammed, Rym Ben Othman, Hamid Alghurabi, Radhwan M. Hussein, Zaid Al-Obaidi, Haifa Abdesselem

**Affiliations:** a University of Monastir, College of Pharmacy, Tunisia; b Department of Clinical Pharmacy, University of Kerbala, College of Medicine, Kerbala, Iraq; c University of Tunis el Manar, Faculty of Medicine of Tunis, Tunisia; d Institut National de Nutrition et de Technologie Alimentaire de Tunis, Tunisia; e Department of Drug Delivery and Nano Pharmaceutics, Graduate School of Pharmaceutical Sciences, Nagoya City University, Nagoya, Japan; f Department of Pharmaceutics and Clinical Lab. Sciences, College of Pharmacy, Ahl Al Bayt University, Kerbala, Iraq; g Department of Biosciences, College of Health and Life Sciences, Aston University, Birmingham, UK; h Department of Physiology, College of Medicine, University of Kerbala, Karbala, Iraq.

**Keywords:** adhesion molecules (P-selectin, E-selectin), insulin therapy, lipid profile, metformin, pro-inflammatory cytokines (IL-6, TNF-α), type 2 diabetes mellitus

## Abstract

Type 2 diabetes mellitus (T2DM) is a major global health issue associated with chronic inflammation, which contributes to both disease progression and its complications, including cardiovascular and microvascular disorders. Key inflammatory markers such as tumor necrosis factor-alpha, interleukin-6 (IL-6), E-selectin, and P-selectin are elevated in T2DM patients and are implicated in the development of these complications. Understanding how treatments such as insulin and metformin affect these markers is crucial for improving therapeutic strategies in T2DM. This study investigated the effects of insulin and metformin on these inflammatory markers in T2DM patients. This was a cross-sectional study involving patients with diabetes on insulin (group A), metformin only (group B), and healthy controls (group C). Participants were enrolled from the Diabetic Center in Karbala, Iraq and underwent clinical assessments including ophthalmologic examinations. Fasting blood glucose, HbA1c and lipids levels were assessed. The levels of inflammatory markers (IL-6 and TNF-α), and adhesion molecules (sE-selectin and sP-selectin) were measured using Enzyme-Linked Immunosorbent Assay (ELISA). The study included 522 patients with diabetes: 356 receiving insulin (group A), 70 receiving metformin (group B) and 96 healthy controls (group C). T2DM patients treated with insulin exhibited significantly more microvascular complications than those treated with metformin. Higher rates of retinopathy (64.3% vs 11.4%) and neuropathy (69.9% vs 11.4%) were observed in the insulin group, whereas the incidence of nephropathy did not differ significantly (14.6% vs 11.4%). Inflammatory markers were lower in the insulin group: TNF-α levels were 3-fold lower and IL-6 levels were 8-fold lower. Conversely, sE-selectin levels were 1.5-fold higher in the insulin group, and sP-selectin levels were 1.4-fold higher in the metformin group. This study highlights distinct differences in inflammatory markers and systemic complications between T2DM patients treated with insulin and those treated with metformin alone. Further research is needed to explore the mechanisms underlying these observations and optimize treatment strategies for T2DM patients.

## 1. Introduction

Type 2 diabetes mellitus (T2DM) is a major global health issue affecting different body systems. It is an important public health issue worldwide for that reason, with forecasts indicating that by 2050, more than one-third of the world’s population will be affected. Recent studies have found approximately 46 million T2DM patients in the Middle East.^[1]^

T2DM is a determining factor of microvascular complications such as nephropathy, retinopathy, neuropathy and small vessel vasculopathy, which can result in lower extremity amputation.^[2]^ Diabetic nephropathy is responsible for 38.6% of new cases of end-stage renal disease while diabetic retinopathy is 1 of the causes of 11.7% of vision disabilities among diabetic patients.^[3]^

Recent research has shown that low-grade inflammation may be involved in the pathogenesis of T2DM.^[4]^ A substantial long-term effort has been dedicated to investigating the relationship between different circulating inflammatory markers and parameters of diabetes.^[5–8]^

Among the pro-inflammatory cytokines are IL-6 and TNF-α^[9]^ as well as adhesion molecules including soluble E-selectin (sE-selectin)^[10]^ and P-selectin.^[11]^ Adhesion of leukocytes to endothelial cells is a critical early step in the development of vascular complications. These adhesion molecules play a pivotal role in mediating inflammation, endothelial dysfunction, and progression of both microvascular and macrovascular complications. This process occurs through sequential steps regulated by specific adhesion molecules present on the leukocytes and endothelial cells. Selectins, specifically E-selectin and P-selectin are among the key adhesion molecules involved in the development of microvascular complications. Evidence indicates that the levels of these cell adhesion molecules are altered in patients with T2DM who experience microvascular complications such as neuropathy, retinopathy, and nephropathy. Furthermore, early detection of these altered levels in the bloodstream can serve as a predictive marker for the development of such complications.^[11]^

Chronic inflammation is a hallmark of T2DM, contributing to both the development of the disease and its complications. In particular, pro-inflammatory cytokines, such as tumor necrosis factor-alpha (TNFA) and interleukin-6 (IL-6), along with adhesion molecules like E-selectin and P-selectin, play a crucial role in mediating the inflammatory responses associated with T2DM and its cardiovascular and metabolic complications. Previous studies have shown that these markers are also linked to broader inflammatory diseases, such as thrombosis and vascular inflammation.^[12]^

In patients with T2DM, elevated levels of TNFA, IL-6, E-selectin, and P-selectin are frequently observed, reflecting the increased burden of systemic inflammation associated with the disease.^[13]^ These markers are closely linked to the development of diabetic complications, including cardiovascular disease and microvascular damage.^[13]^ Moreover, recent research highlights the role of these inflammatory mediators in exacerbating metabolic disturbances in T2DM patients.^[14,15]^ Given this evidence, studying the effects of insulin and metformin on these key inflammatory markers is critical for understanding their broader therapeutic impact in managing inflammation in T2DM patients.

The objective of this study was to evaluate and compare inflammatory markers, glycemic control, lipid profiles and the incidence of microvascular complications in T2DM patients treated with insulin versus those treated with metformin alone and a control group.

## 2. Methodology

### 2.1. Study design

**Figure 1.**
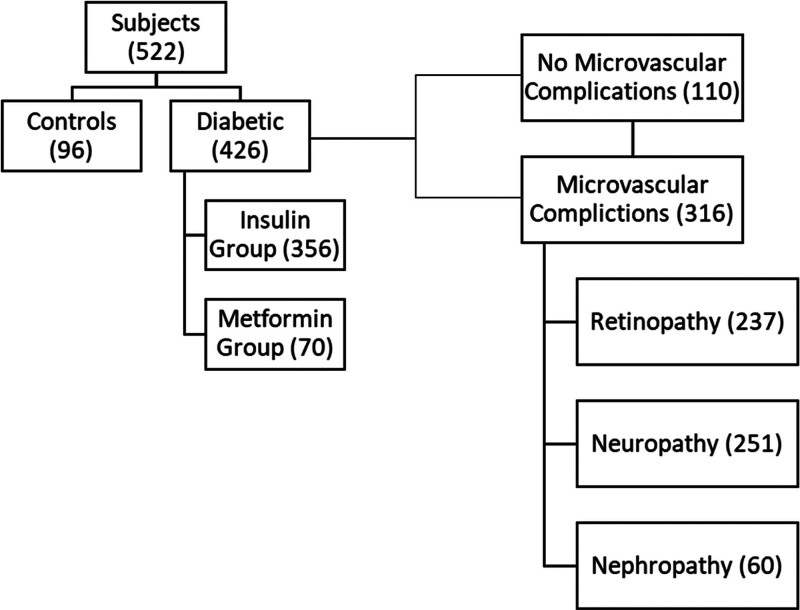
Distribution of subjects and microvascular complications among diabetic patients.

The study was performed on 522 patients with diabetes, of which 356 patients were treated with insulin (group A) and 70 patients were treated with metformin (group B). In addition, 96 healthy individuals (group C) with no history of diabetes or chronic inflammatory diseases were included in the study to provide baseline reference values for the inflammatory markers, though direct comparisons with the diabetic groups were not the primary focus of the analysis (Fig. 1). Of the total population, 280 were females and 242 were males.

### 2.2. Inclusion and exclusion criteria

Participants in this study were adults aged 18 years or older with a confirmed diagnosis of T2DM based on the American Diabetes Association criteria. Eligible patients had been receiving either insulin or metformin monotherapy for at least 6 months before the study commenced. Additionally, healthy control individuals, aged 18 or older with no history of diabetes or chronic inflammatory diseases, were included for comparative purposes.

Exclusion criteria included patients with a diagnosis of type 1 diabetes mellitus, gestational diabetes, or those on combination therapy involving both insulin and metformin or other glucose-lowering medications. Patients who had been using immunosuppressive drugs, corticosteroids, or any other medications known to influence inflammatory markers within 3 months prior to the study were also excluded. Other exclusion criteria included pregnancy, lactation, active infections, autoimmune diseases, malignancies, or any condition that could significantly affect inflammatory marker levels.

### 2.3. Study population

Participants were recruited from the Diabetic Center in Karbala, Iraq. Patients were grouped according to clinical evaluations, including, but not limited to, ophthalmological examinations. The retinopathy group was assessed by an ophthalmologist, neuropathy group by a specialist neurologist, and nephropathy group based on the estimated glomerular filtration rate (eGFR). Of the 426 T2D’ patients analyzed, 237 (45.4%) had diabetic retinopathy, 251 (48.1%) had diabetic neuropathy, 60 (11.5%) had nephropathy, and 316 (60.5%) had microvascular complications.

### 2.4. Participants screening

Participants were asked to fill out a questionnaire that obtained sociodemographic information such as age, gender, occupation, and place of residence. Thorough medical histories were also provided, including the duration of the disease, treatment methods, history of eye diseases, renal examination laboratory results, and neuropathy examination results. The participants were subsequently divided into 2 groups based on their treatment: insulin or oral hypoglycemic agents. In addition, a general clinical examination, including the measurement of blood pressure, was performed on all individuals.

### 2.5. Anthropometric measurements

Height and weight were measured to calculate the body mass index (BMI) using the following formula: weight in kilograms divided by height in meters squared. Based on their BMI, patients were divided into 3 groups: normal weight (BMI < 24.9 kg/m²), overweight (BMI 25–29.9 kg/m²), and obese (BMI > 30 kg/m²).^[16]^

### 2.6. Laboratory measurement

All subjects underwent glycemic control testing in terms of fasting blood glucose and HbA1c levels. Additionally, fasting serum lipid levels were measured, which required a fasting period of 8 hours.

The participants were categorized into 2 groups based on their HbA1c levels: controlled (<7%) and uncontrolled (≥7%). According to the American Diabetes Association guidelines, abnormal serum lipid levels were defined as follows: total cholesterol ≥ 200 mg/dL, low-density lipoprotein ≥ 130 mg/dL, high-density lipoprotein < 40 mg/dL, and triglycerides ≥ 150 mg/dL.^[17]^ HbA1c and lipid assays were performed using a chemistry analyzer (Cobas Integra 400 Plus, Roche Diagnostics, Mannheim, Germany). The levels of IL-6, TNF-α, E-selectin, and P-selectin were determined using a quantitative sandwich enzyme-linked immunosorbent assay (ELISA; R&D Systems, Minneapolis). eGFR was calculated using the following formula: Cr is serum creatinine, e GFR (mL/min/1.73 m^2^) = 194 × Cr ^−1094^ × Age ^−0.287^ (* 0.739 if female).^[18]^

### 2.7. Ethical consideration

This study was conducted in accordance with the ethical principles outlined in the Declaration of Helsinki. Prior to enrollment, all participants provided informed consent after being fully informed of the study’s purpose.

The ethical committee of the College of Medicine at the University of Kerbala approved this study (approval number 22) on April 12, 2022. All participants provided written informed consent before enrollment in the study.

### 2.8. Statistical analysis

Statistical analyses were conducted using the IBM SPSS Statistics software (version 26.0; IBM Corporation, Armonk). Continuous variables were reported as either mean ± standard deviation or median (interquartile range) and were analyzed using either the Student *t* test or Mann–Whitney *U* test, depending on the data distribution. The normality of continuous variables was tested using the Shapiro–Wilk or Kolmogorov–Smirnov tests, depending on the sample size, and further assessed by visual inspection of Q-Q plots. Categorical variables are presented as frequencies and percentages and were compared using chi-square tests. An Analysis of Covariance was used to assess the effect of treatment group on inflammatory markers while controlling for disease duration. Statistical significance was set at *P* of <.05.

## 3. Results

Table 1 summarizes the demographic and clinical characteristics of the study participants. T2DM patients were significantly older, had a higher BMI, and included fewer men compared to the control group.

**Table 1:** Demographic and clinical characteristics of the study participants.

	Controls (n = 96)	T2DM patients (n = 426)	*P*-value
Gender, male	63 (65.5%)	179 (42%)	<.001
Age (yr)	46.1 ± 9.64	55.5 ± 8.92	<.001
Body mass index (kg/m^2^)	25.3 ± 1.28	30 ± 5.39	<.001

Note: Continuous variables are expressed as mean ± SD, while categorical variables are expressed as frequency (percentage).

The demographic and clinical characteristics of T2DM patients treated with insulin and metformin are summarized in Table 2. Patients on insulin were older and had higher levels of low density lipoprotein cholesterol, triglycerides, fasting blood glucose, HbA1c, and creatinine but lower levels of total cholesterol, high density lipoprotein (HDL) cholesterol, and eGFR. In addition, these patients had longer disease durations. BMI and proportion of men were comparable between the 2 groups.

**Table 2:** Demographic and clinical characteristics of T2DM patients treated with insulin and those treated with metformin.

	Insulin group (n = 356)	Metformin group (n = 70)	*P*-value
Gender, male	153 (43%)	26 (37.1%)	.366
Age (yr)	56.2 ± 8.66	52.2 ± 9.54	<.001
Body mass index (kg/m^2^)	29.8 ± 5.46	30.6 ± 4.97	.259
eGFR (mL/min)	90.9 ± 31.6	101 ± 29.7	.013
Creatinine (mg/dL)	0.82 ± 0.27	0.74 ± 0.24	.023
Fasting blood glucose (mg/dL)	267 ± 110	205 ± 73.3	<.001
HbA1c (%)	9.94 ± 1.91	8.40 ± 1.76	<.001
Total cholesterol (mg/dL)	196 ± 49.9	215 ± 32.3	<.001
HDL cholesterol (mg/dL)	38.5 ± 6.73	40.5 ± 9.87	.039
LDL cholesterol (mg/dL)	120 ± 20.1	114 ± 20.8	.026
Triglycerides (mg/dL)	191 ± 92.4	175 ± 52.3	.048
Duration of disease (yr)	13.2 ± 6.47	6.40 ± 4.94	<.001

Continuous variables are expressed as mean ± SD, while categorical variables are expressed as frequency (percentage).

HDL = high density lipoprotein, LDL = low density lipoprotein.

**Figure 2.**
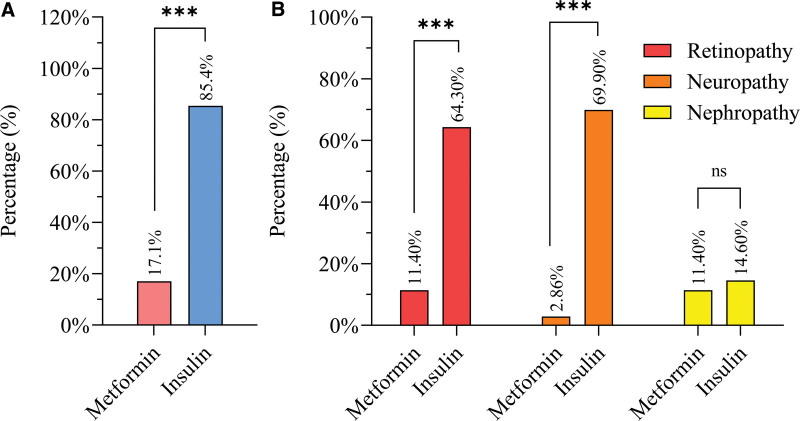
(A) Overall and (B) detailed Microvascular complications in T2D patients that take insulin (n = 356) and T2D patients that take metformin (n = 70). Statistical significance is indicated by (ns *P* ≥ .05, ***P < .001).

Notably, T2DM patients treated with insulin exhibited significantly more microvascular complications than those treated with metformin. Specifically, higher rates of retinopathy (64.3%) and neuropathy (11.4%) were observed in the insulin group (69.9%) in the metformin group (11.4%). However, the incidence of nephropathy did not differ significantly between the 2 groups (insulin group, 14.6% vs metformin group, 11.4%) (Fig. 2).

**Figure 3.**
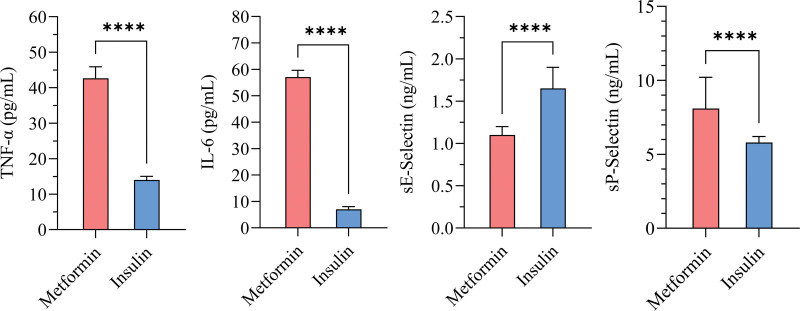
Levels of inflammatory markers and circulating endothelial adhesion molecules in T2D patients treated with insulin (n = 356) versus those treated with metformin (n = 70). Data are expressed as median (25th–75th percentile). Statistical significance is indicated by (****P < .0001).

T2DM patients treated with insulin had lower levels of TNF-α (3 folds), IL-6 (8 folds), and sP-selectin (1.4 folds) compared to those treated with metformin (Fig. 3). Conversely, T2DM patients receiving insulin exhibited significantly higher levels of sE-selectin (1.5 folds).

An analysis of covariance was conducted to examine the effect of treatment group (metformin vs insulin) on TNFA, IL-6, E-selectin, and P-selectin, while controlling for disease duration. The results in (Table 3) showed that the treatment group had a significant effect on all 4 inflammatory markers, with large effects observed for TNFA and IL-6, and moderate effects for E-selectin and P-selectin. In contrast, disease duration did not have a significant impact on any of the markers.

**Table 3:** The analysis of covariance (ANCOVA) results for TNFA, IL-6, E-selectin, and P-selectin levels by treatment group, controlling for disease duration.

	Factor	df	*F*	*P*-value	Partial eta squared
TNFA (pg/mL)	Treatment group	(1, 423)	2291.368	<.001	0.844
	Disease duration	(1, 423)	0.343	.558	
IL-6 (pg/mL)	Treatment group	(1, 423)	13,767.714	<.001	0.970
	Disease duration	(1, 423)	0.902	.343	
E-selectin (ng/mL)	Treatment group	(1, 163)	47.378	<.001	0.225
	Disease duration	(1, 163)	0.033	.856	
P-selectin (ng/mL)	Treatment group	(1, 163)	28.130	<.001	0.147
	Disease duration	(1, 163)	0.790	.376	

Note: ANCOVA was conducted to assess the effects of treatment group (metformin vs insulin) on TNFA, IL-6, E-selectin, and P-selectin, with disease duration included as a covariate.

## 4. Discussion

Assessment of inflammatory markers and their implications for atherogenic risk are critical in patients with T2DM receiving insulin. This study was designed to investigate the relationship between pro-inflammatory cytokines and adhesion molecules, specifically by comparing the levels of P-selectin, IL-6, and TNF-α in patients treated with insulin versus those treated with metformin.

This study demonstrated a significant reduction in P-selectin, marking the first human study to observe this effect, especially when compared with metformin.

Martin et al^[19]^ showed that insulin treatment modulates the inflammatory response by regulating the production of inflammatory markers and expression of P-selectin in the lungs. It is observed that the current finding is in agreement with the previous 1. Furthermore, insulin treatment significantly reduced the levels of inflammatory cytokines (IL-6 and TNF-α) in T2DM patients, as previously demonstrated in clinical studies. This anti-inflammatory effect of insulin is attributed to the release of nitric oxide and expression of endothelial nitric oxide synthase. This leads to vasodilation and reduced leukocyte adhesion to the endothelium, thereby exerting anti-inflammatory effects.^[20–25]^

Selectins, the adhesion molecules on cells, are indispensable for the mobilization of white blood cells and for the activation of endothelial cells. They also play a key role in the inflammatory cascade, which is responsible for the vascular complications in T2DM. Modulation of P-selectin levels in patients with T2DM is a pivotal factor in vascular inflammation and dysfunction. These outcomes are in line with those of previous studies showing elevated plasma levels of soluble adhesion molecules in diabetes mellitus.^[10,26]^

T2DM is associated with increased levels of acute-phase response markers and IL-6 in blood. An enhanced acute-phase response may contribute to the many clinical and biochemical features of T2DM and its complications. This study aimed to confirm the elevated circulating concentrations of the acute-phase mediators IL-6 and TNF-α in T2DM and to investigate blood as a potential source of these cytokines.^[9,27]^ Researchers, including Raskin et al, have implicated cytokines such as TNF-α and IL-6 in the pathogenesis of T2DM.^[28]^ Previous studies have shown that long-term insulin treatment in T2DM not only improves hyperglycemia but also significantly decreases pro-inflammatory mediators (TNF-α and IL-6).^[22]^ Additionally, insulin significantly increased the levels of anti-inflammatory cytokines, such as IL-10 and IL-4. Recent findings have indicated that extended insulin administration notably reduces pro-inflammatory cytokine levels in patients with T2DM.

Elevated plasma levels of soluble adhesion molecules in T2DM have been well-documented.^[10,29,30]^ Moreover, a positive correlation was observed between dyslipidemia and the presence of E-selectin.^[10,31]^

The results showed that patients with T2DM receiving insulin had a higher level of E-selectin than those who were treated with metformin, although the increase was not significant. These findings were consistent with those reported by Leena et al.^[32]^

Disease duration did not significantly affect any of the inflammatory markers. This suggests that the observed differences in marker levels are primarily driven by the type of treatment rather than the length of time the patients have had diabetes. These results are consistent with previous studies highlighting the anti-inflammatory effects of Metformin. However, further research is needed to explore the long-term implications of these findings and whether they translate to clinical benefits.

To evaluate the effectiveness of glycemic control in patients with T2DM, it is crucial to estimate mortality based on GFR. Yamaguchi et al^[33]^ revealed a drop in eGFR when administering insulin to patients. This finding is in line with the statistical analysis of the results of the current study, which showed that there was a decrease in the eGFR of patients with T2DM who received insulin compared with those who received metformin. This decrease in eGFR demonstrates the potential of insulin treatment to constrain nephron function in patients with T2DM.

A study of the demographic characteristics of T2DM patients who were treated with insulin and those treated with metformin showed a statistically significant difference in renal function. Insulin therapy was linked to a longer duration of diabetes, a greater HbA1c level, and an increased fasting blood glucose level (Table 2). Moreover, insulin-treated patients showed better lipid profiles and BMIs. Insulin treatment in T2DM patients was associated with a higher high density lipoprotein cholesterol level, a lower low density lipoprotein cholesterol level, and a different total cholesterol level than metformin treatment. Previous research has indicated that individuals with T2DM who use insulin tend to have a more favorable lipid profile than those who use metformin.^[9,34]^

T2DM has more complications that are proportional to the duration of the disease. Diabetic retinopathy is a prognostic state that leads to the destruction of pancreatic B-cell function, which causes a decrease or absolute loss of insulin inside the body. At this stage, patients require insulin therapy to maintain health. The current study demonstrated significant retinopathy in with patients receiving insulin treatment compared to metformin in T2DM. Indeed, this finding is in agreement with those of previous studies.^[35–37]^

The novel finding of reduced P-selectin levels in T2DM patients in the current study paves the way for further research into the relationship between insulin treatment and biomarkers, particularly adhesion molecules. This research should extend beyond metabolic effects on renal function, BMI, HbA1c, fasting blood glucose, and lipid profiles in T2DM patients.

These results reinforce the potential anti-inflammatory benefits of metformin, which go beyond glycemic control and may influence long-term outcomes in T2DM patients. In clinical practice, these findings support the consideration of metformin as a first-line treatment, not only for its glucose-lowering effects but also for its capacity to reduce inflammation and potentially prevent the progression of diabetic complications. Conversely, the use of insulin may require careful monitoring of inflammation markers and vascular health, especially in patients with prolonged disease duration or existing complications.

It should be noted that while all participants had been receiving insulin or metformin monotherapy for at least 6 months prior to the study, detailed data on the exact duration of treatment beyond this period were not collected. This limitation prevents the assessment of whether longer treatment durations could have had a more pronounced effect on the inflammatory markers and T2DM complications. Future research should include treatment duration data to explore its potential impact on the results.

## 5. Conclusion

The results showed that insulin treatment for T2DM significantly reduced the P-selectin levels. This is a unique effect of insulin treatment on P-selectin reduction, a rare occurrence in vitro and seldom in human studies. In addition to reducing pro-inflammatory cytokines (IL-6 and TNF-α), insulin treatment also exerts a metabolic effect that enhances lipid profiles compared to metformin therapy. This finding supports the use of a comprehensive approach to managing T2DM that considers the complex interplay between inflammation, atherosclerosis, and the mitigation of progressive complications.

### 5.1. Limits of the study

The cross-sectional design restricts the possibility of deducing the causative relationship between insulin and metformin treatment and their impact on inflammatory markers and microvascular complications. The study was conducted in a single center in Karbala, Iraq, which could be the reason why the results of this study apply to a narrower range of people. Self-reported data may have introduced recall bias. The study did not control for possible confounders, such as diet and physical activity. Moreover, inflammatory markers were recorded at only 1 time point, which may not be sufficient to indicate that they were stable over time. Future longitudinal studies with larger and more diverse populations are necessary to confirm our results.

Another limitation of this study is the absence of data on comorbid chronic diseases and the use of certain medications that could influence inflammatory markers, such as statins and nonsteroidal anti-inflammatory drugs (NSAIDs). Although efforts were made to exclude patients on corticosteroids or immunosuppressive therapies, the influence of other medications or underlying conditions cannot be fully ruled out. This lack of data on comorbidities and medication use may have introduced confounding factors in the analysis of cytokine and adhesion molecule levels. Future studies should include detailed tracking of comorbidities and concurrent medication use to better isolate the effects of insulin and metformin on inflammatory markers in patients with T2DM, providing a more comprehensive understanding of these relationships.

## Acknowledgments

We extend our gratitude to the patients for their participation and to the medical staff at the AL-Hassan Metabolism, Endocrine, and Diabetes Center, Karbala Governorate, Iraq, for their invaluable support.

## Author contributions

**Conceptualization:** Rym Ben Othman.

**Data curation:** Haithem Mohammed, Hamid Alghurabi, Radhwan M. Hussein.

**Formal analysis:** Haithem Mohammed, Hamid Alghurabi.

**Funding acquisition:** Haithem Mohammed.

**Investigation:** Haithem Mohammed.

**Methodology:** Haithem Mohammed.

**Project administration:** Haithem Mohammed, Zaid Al-Obaidi.

**Resources:** Haithem Mohammed, Hamid Alghurabi.

**Software:** Hamid Alghurabi.

**Supervision:** Rym Ben Othman, Haifa Abdesselem.

**Validation:** Haithem Mohammed, Hamid Alghurabi.

**Visualization:** Hamid Alghurabi.

**Writing – original draft:** Haithem Mohammed.

**Writing – review & editing:** Rym Ben Othman, Hamid Alghurabi, Zaid Al-Obaidi.
